# Gendered Dyadic Coping and Marital Satisfaction Among Chinese Couples Navigating Mild Cognitive Impairment

**DOI:** 10.1093/geroni/igaf122.2854

**Published:** 2025-12-31

**Authors:** Dexia Kong

**Affiliations:** The Chinese University of Hong Kong, Hong Kong, Hong Kong, China (People’s Republic)

## Abstract

**Background:**

This study investigates how dyadic coping (i.e., couples’ joint efforts to manage life stressors) interacts with gender and gender ideologies to influence marital satisfaction among couples living with mild cognitive impairment (MCI) in Hong Kong.

**Methods:**

Using cross-sectional data from 202 couples living with MCI, we applied actor–partner interdependence moderation models to examine interactions between dyadic coping, ideological (dis)similarity, and the gender of the spouse with MCI.

**Results:**

Our results reveal significant gendered asymmetries. The wife’s dyadic coping was related to her own marital satisfaction, while the husband’s dyadic coping was not. Dyadic (dis)similarity in gender ideologies matters: Egalitarian wives with MCI reported higher marital satisfaction when their traditionalist husbands engaged in dyadic coping, whereas dyadic coping of traditionalist husbands with MCI diminished their egalitarian wives’ marital satisfaction. Two-way interactions demonstrated that when persons with MCI endorse more traditional ideologies than their spouses, their dyadic coping enhanced their own satisfaction, regardless of gender.

**Conclusions:**

Dyadic (dis)similarity in gender ideologies and gender combinations of MCI-affected couples intersect to shape the dyadic coping-marital satisfaction link. The positive relational impact of dyadic coping is contingent upon deeply rooted Confucian traditions. Reconciling the gap between collaborative practices and patriarchal norms has the potential to mitigate the gendered imbalance in spousal care. The findings provide valuable insights into the development of culturally sensitive dyadic interventions to promote relational outcomes among couples navigating health challenges beyond MCI. The findings extend dyadic health theory and research in a non-Western context.

